# Die Pestarztmaske im Deutschen Medizinhistorischen Museum Ingolstadt

**DOI:** 10.1007/s00048-020-00255-7

**Published:** 2020-05-25

**Authors:** Marion Maria Ruisinger

**Affiliations:** 1 Deutsches Medizinhistorisches Museum Ingolstadt, Anatomiestr. 18–20, 85049 Ingolstadt, Deutschland; 2 Friedrich-Alexander-Universität Erlangen-Nürnberg, Erlangen, Deutschland

**Keywords:** Pestmaske, Schutzkleidung, materielle Kultur, Ikonographie, Museologie, plague mask, protective clothing, material culture, iconography, museology

## Abstract

Dieser Beitrag ist Teil des Forums COVID-19: Perspektiven in den Geistes- und Sozialwissenschaften. Die Figur des Pestarztes mit der schnabelförmigen Maske ist heute die am häufigsten zitierte Bildmetapher für die Pest. Es verwundert daher nicht, dass die Pestarztmaske in der Sammlung des Deutschen Medizinhistorischen Museums in Ingolstadt zu den am meisten nachgefragten Objekten und Bildmotiven des Hauses gehört. Der Forumsbeitrag spürt der Figur des Pestarztes auf mehreren Ebenen nach: Zunächst wird anhand zeitgenössischer Text- und Bildquellen diskutiert, welche Art von Schutzkleidung zu Seuchenzeiten empfohlen wurde und welche Rolle das schnabelförmige Maskenattribut dabei spielte. Anschließend wird das Ingolstädter Exemplar unter die Lupe genommen, hinsichtlich seines Materials und seiner Machart auf Authentizität und Praktikabilität hin untersucht und mit dem Exemplar des Deutschen Historischen Museums in Berlin verglichen.

Das Ergebnis: Die frühesten Belege für die Verwendung dieser speziellen Art von Schutzkleidung stammen aus Italien und Südfrankreich und datieren aus der Mitte des 17. Jahrhunderts. Für Mitteleuropa gibt es keinerlei Nachweise dafür, dass zu Pestzeiten jemals eine solche Schnabelmaske in Gebrauch gewesen wäre. Und die Pestarztmasken in Ingolstadt und Berlin? Bei kritischer Betrachtung finden sich bei beiden Masken Details, die gegen eine Verwendung als Schutzkleidung sprechen. Ob es sich dabei um ältere, historisierende Nachbauten oder bewusste Fälschungen handelt, muss offen bleiben.

**Zusatzmaterial online:**

Zusätzliche Informationen sind in der Online-Version dieses Artikels (10.1007/s00048-020-00255-7) enthalten.

## Abstract (en)

This paper is part of Forum COVID-19: Perspectives in the Humanities and Social Sciences. The figure of the plague doctor with the beak mask has become the symbol of the plague par excellence. It’s little wonder that the plague mask in the collection of the German Museum of the History of Medicine in Ingolstadt (Bavaria) is one of the museum’s most popular objects and motifs. This forum paper investigates the figure of the plague doctor on several levels: first, it analyses contemporary textual and image sources in regard to protective clothing used in times of plague and the respective role of the beak-like part of the mask. Then it takes a close look at the Ingolstadt specimen. By examining the mask’s materiality and fabrication, questions of its authenticity and practicability are raised. Finally, the Ingolstadt mask is compared with the specimen at the German Historical Museum in Berlin.

The conclusion: the beak mask is not mentioned before the mid-seventeenth century, and then only in Italy and Southern France. There is no proof at all of its use during plague outbreaks in Middle Europe. And the specimens in Ingolstadt and Berlin? Both masks present details which suggest that they were not used as protective clothing at all. We do not know, however, if they were produced as replicas for historic reasons or as fakes for the modern art market.

Die Leiterin der Kulturredaktion einer schwäbischen Tageszeitung wünschte Anfang April 2020 Bildmaterial zu einem Interview, in dem ich – wie so viele Kolleginnen und Kollegen – während der Covid-19-Pandemie zur Geschichte der Seuchen befragt worden war. Sie äußerte dabei auch schon einen konkreten Wunsch: „die Pestmaske“. Auf meine etwas irritierte Nachfrage hin, warum sie denn gerade von diesem Objekt ein Foto bringen wolle, ich hätte es doch im Interview gar nicht erwähnt, war die Antwort: „Da haben Sie recht, dann lassen wir das mit der Pestmaske (das sieht halt immer so spektakulär aus).“

Dies ist beileibe kein Einzelfall. Die Pestarztmaske mit der Inventarnummer 02/222 (Abb. 1) gehört zu den beliebtesten Objekten des Deutschen Medizinhistorischen Museums in Ingolstadt (DMMI) – und das nicht nur bei der Presse und unseren Museumsgästen, sondern auch bei der Fachöffentlichkeit.^1^ Immer wieder erreichen uns diesbezügliche Anfragen – von der Lehrerin, die sich die Maske für eine Unterrichtsprobe ausleihen will, über den Mediävistikprofessor, der um eine Reproduktion für einen Quellenband mittelalterlicher Texte bittet, bis hin zu Darstellern auf Historienspektakeln, die sich ein Schnittmuster zum Nachbauen der Maske wünschen. Die meisten Anfragen aber kommen von Kolleginnen und Kollegen aus der europäischen Museumswelt, die unsere Maske – sei es als digitale Reproduktion, als handwerklich gefertigte Replik oder im Original – für eigene Ausstellungszwecke verwenden wollen. Die historischen Pestausbrüche, zu deren Illustration die Maske jeweils dienen soll, datieren mal ins Mittelalter, mal in den Dreißigjährigen Krieg, mal ins frühe 18. Jahrhundert, ja selbst für eine Ausstellung zur Geschichte des Wetters wurde sie schon gewünscht.

**Figure 1:**
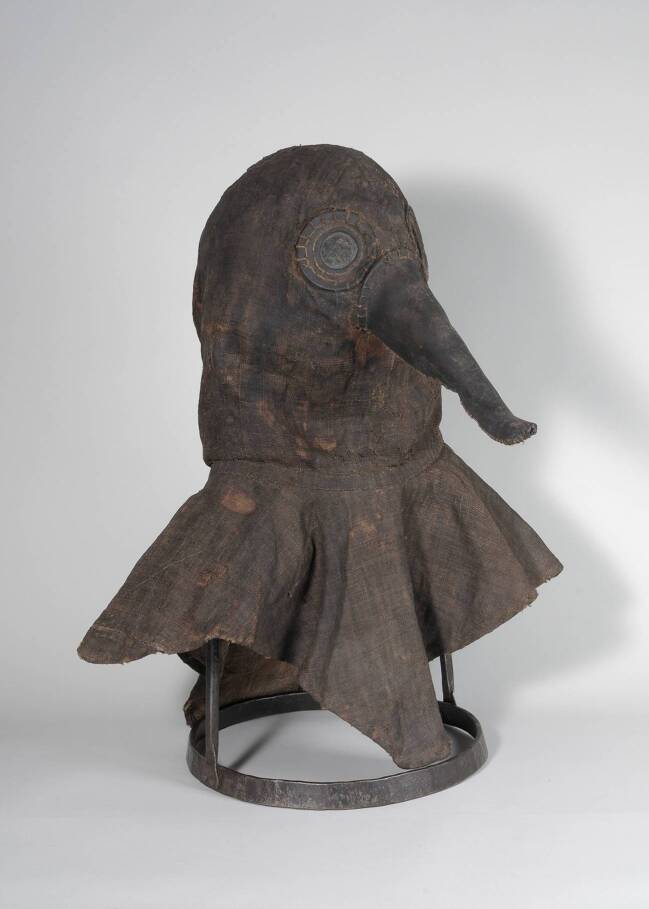

Den diversen Anfragen pflegen wir mit der immer gleichen Gegenfrage zu antworten: „Können Sie belegen, dass in der betreffenden historischen Situation eine Pestarztmaske getragen wurde?“ Daraus entwickeln sich oft interessante Gespräche; bislang mussten jedoch alle Anfragen abschlägig beschieden werden, weil in keinem Fall die Verwendung dieser Schutzkleidung nachgewiesen werden konnte.

Offensichtlich ist das Bild des Pestarztes mit der Schnabelmaske heute in so hohem Grad ikonisch für „die Pest“, dass selbst Fachleute es nicht weiter hinterfragen, sondern die vom Publikum herangetragene Erwartungshaltung bereitwillig bedienen oder gar selbst der suggestiven Skurrilität des Pestarztes erliegen. Und so gehört der Schnabeldoktor heute zu den selbstverständlichen Statisten musealer Pest-Inszenierungen^2^ ebenso wie zu den beliebtesten Titelmotiven seuchenhistorischer Literatur (z. B. Leven 1997; Ulbricht 2002; Eikermann & Kaiser 2012; Jacobsen 2012). Die Figur des Pestarztes gilt uns heute als die Bildmetapher für die Pest schlechthin. Aber entspricht dies auch den historischen Gegebenheiten? Wie bedeutend war diese spezielle Form der Schutzkleidung in Seuchenzeiten tatsächlich? Und – um die Fragestellung auf das Exemplar des DMMI engzuführen – ist unsere Pestarztmaske „echt“?

Im Folgenden stelle ich die ärztliche Kleidung in Seuchenzeiten im Allgemeinen und die Pestarztmaske im Besonderen zunächst auf Basis von textlichen Quellen dar, um mich anschließend dem Exemplar der Ingolstädter Sammlung und einem Vergleichsexemplar aus dem Deutschen Historischen Museum in Berlin zuzuwenden.

## Schutzkleidung in Seuchenzeiten: Material und Machart

Eine ärztliche Berufskleidung im eigentlichen Sinn entstand erst als Reaktion auf die bakteriologischen Erkenntnisse gegen Ende des 19. Jahrhunderts. Vorher kleidete sich ein *Medicus* (akademisch ausgebildeter Arzt) oder *Chirurgus *(handwerklich ausgebildeter Wundarzt) so, wie es seinem jeweiligen, meist bürgerlichen, Stand entsprach. Allerdings galt seit der Antike die Regel, dass Ärzte und Wundärzte durch ein sauberes, gepflegtes Erscheinungsbild und angenehme Umgangsformen das Vertrauen ihrer Klientel erwerben sollten (Castro 1614: 124–127; Heister 1719: 14). In Seuchenzeiten galten andere Prioritäten. Für den Arzt war es nun wichtiger, sich beim Krankenbesuch vor Ansteckung zu schützen, als durch gefällige Kleidung einen guten Eindruck im Hause der Kranken zu hinterlassen.

In der vorbakteriologischen Zeit gab es im Wesentlichen zwei Erklärungen für das Phänomen der Ansteckung: Eine Krankheit konnte durch ein stofflich gedachtes Krankheitsgift (*contagium*) oder durch verdorbene Luft (den „Pesthauch“) übertragen werden. Als probates Mittel zur Vertreibung des Pesthauchs aus der Krankenstube galten Feuer und Räucherungen. Die individuelle Atemluft konnte gereinigt werden, indem man sich einen mit Duftessig getränkten Schwamm oder ein Säckchen mit in Essig getränkten aromatischen Kräutern vor die Nase hielt, wenn man sich dem Kranken näherte (Stockhammer 1713: 61). Für die Kleidung des Arztes wurden lange, an den Handgelenken geschlossene Ärmel empfohlen, damit die Krankheitsstoffe nicht unter das Gewand eindringen konnten. Ein möglichst glatter Stoff sollte verhindern, dass sie an der Oberfläche haften blieben. Als geeignet galt etwa gepresstes Leinen, das zusätzlich durch eine Behandlung mit Öl oder Wachs abgedichtet werden konnte. Diese Schutzprinzipien wurden auch bei der Epidemie von 1720 in Marseille befolgt, wie dem Bericht Johann Jacob Scheuchzers zu entnehmen ist, der sich auf Mitteilungen der dort tätigen Ärzte bezieht:

Der Kleideren halb hat man sich zu hüten vor allem, was auß Tuch, oder Baumwolle gemachet wird, weilen das Gifft sich leicht an dergleichen Sachen henket. Besser sind die leinernen, seidenen, tafteten Kleider, oder von Cameel-Haaren, noch besser, sonderlich vor die, so um die Kranken seyn müssen, dicht lederne, oder gar von Wachs- und Harz-Tuch, welche von denen Marsilianischen Doctoribus sollen gebraucht worden seyn. Alle Kleider aber sollen reinlich gehalten, offt abgeänderet, zuweilen beräucheret, und in freye Lufft gehenket werden. (Scheuchzer 1720: 34)

Diese Empfehlungen galten nicht nur für Ärzte und Chirurgen, sondern auch für die Pestbediensteten, die betroffene Häuser reinigten oder Umgang mit Pestkranken hatten. Beispielsweise sah eine Schlesische Verordnung für diesen Personenkreis „eng anliegende Bekleidung aus gewachster Leinwand und ebensolche Handschuhe“ vor (Schlenkrich 2013: 201).

## Schutzkleidung in Seuchenzeiten: Verhüllung des Kopfes

In den oben angeführten Texten zur ärztlichen Kleidung bei Seuchenzeiten finden sich keine Empfehlungen zur Verhüllung des Hauptes. An anderer Stelle gibt es jedoch Hinweise darauf, dass Ärzte und Pestbedienstete im 17. Jahrhundert ihre Schutzkleidung gelegentlich durch eine Kopfhaube zu ergänzen pflegten. So gibt die 1680 erschienene Pestschrift „Einfältiger Discursus Sanitatis“ eine genaue Anweisung für die Anfertigung einer haubenartigen Kopfbedeckung für Totengräber, Reiniger und Pestbüttner mit Glaseinsätzen vor den Augen (Schlenkrich 2013: 201, Anm. 100).

Auch bei der Elfenbeinstatuette, die 1977 für unser Haus im deutschen Kunsthandel erworben wurde, und die mit ihrer spitz zulaufenden Kopfhaube auf den ersten Blick an einen Vertreter des Ku-Klux-Klans erinnert, soll es sich der Überlieferung zufolge um die Darstellung eines „Arztes mit Pestschutzkleidung“ handeln (Abb. ESM 1^3^; Wilderotter 1995: 128). Diese Interpretation wird zwar durch eine 1826 veröffentlichte Abbildung gestützt, die der Bildlegende zufolge einen „Chirurgien“ der Quarantänestation von Marseille zeigt (Abb. ESM 2; Robert 1826). Damals lag die letzte Pest von Marseille aber bereits rund 100 Jahre zurück. Dass die Elfenbeinfigur bis ins Detail mit dem Kupferstich übereinstimmt, wirft die Frage auf, ob sie nicht sogar nach dieser Vorlage gearbeitet ist und daher nicht als vermeintlich zeitgenössischer Beleg für die Pestarztkleidung herangezogen werden darf.

Das Prinzip der maximalen Verhüllung wurde mitunter auch für die Erkrankten selbst angewandt. Eine kolorierte Federzeichnung in der Nürnberger Chronik des Weinschenks Wolf Neubauer d. J. (gest. 1621) zum Seuchenjahr 1562 dokumentiert eine solche Situation (Abb. ESM 3; Dross 2015: 303–304). Hier ist der Pestkranke, der auf einer Sänfte zu dem vor den Stadttoren gelegenen Pesthaus getragen wird, mit einem schwarzen Überwurf verhüllt. Diese Maßnahme schützte die gesunden Bürger nicht nur vor den schädlichen Dünsten, die der damaligen Überzeugung nach vom Kranken ausgingen, sondern auch vor dessen Anblick und den damit verbundenen heftigen Gemütsbewegungen, die ihrerseits als Gefahr für die Gesundheit galten.

## Schutzkleidung in Seuchenzeiten: der „Schnabel“

Erst im 17. Jahrhundert wurde die ärztliche Schutzkleidung durch ein weiteres Element ergänzt: eine Halterung für Duftstoffe, die direkt vor der Nase platziert wurde. Dadurch sollte zum einen die kontinuierliche Aufbereitung der Atemluft gewährleistet werden, zum anderen musste sich der Arzt nun nicht länger den Riechapfel, Duftschwamm oder Kräuterbeutel vor die Nase halten, sondern hatte beim Krankenbesuch beide Hände frei. Die ersten Überlegungen zu dieser Optimierung sollen auf den Leibarzt Ludwigs XIII, Charles Delorme (1584–1678), zurückgehen (Mollaret & Brossollet 1965: 43–44).

Der früheste bekannte Beleg für die Verwendung eines solchen Nasenfutterals in Seuchenzeiten bezieht sich auf die Epidemie, die 1656 in Rom herrschte. Der dänische Arzt Thomas Bartholin (1616–1680) nahm die ihm aus Rom übersandte Abbildung eines solchen Pestarztes zum Anlass, um in seiner 1661 erschienenen Sammlung anatomischer und medizinischer Merkwürdigkeiten auch die „Kleidung des Arztes in Seuchenzeiten“ zu behandeln (Bartholin 1661: 142–145; Abb. 2). Er referierte zunächst unter Rückgriff auf ältere Autoren über die Kleidung des Arztes im Allgemeinen sowie in Seuchenzeiten, um sich dann der nur wenige Jahre zurückliegenden Pest von Rom zuzuwenden: Die Pestärzte kleideten sich in ein Gewand aus gepresstem Leinen, an dem die Keime der Krankheit nicht leicht haften blieben; in der linken Hand trugen sie einen Stock als Zeichen ihres Amtes und vor dem Gesicht eine Schnabelmaske, die mit schützenden, wohlriechenden Substanzen angefüllt war. Bartholin bezeichnet diese Aufmachung als „singularis habitus“ (einzigartige Gewandung). Die Gestalt des römischen Schnabeldoktors mit dem Stock in der Hand war für den Arzt in Kopenhagen offenbar eine neue, ungewöhnliche Erscheinung.

**Figure 2:**
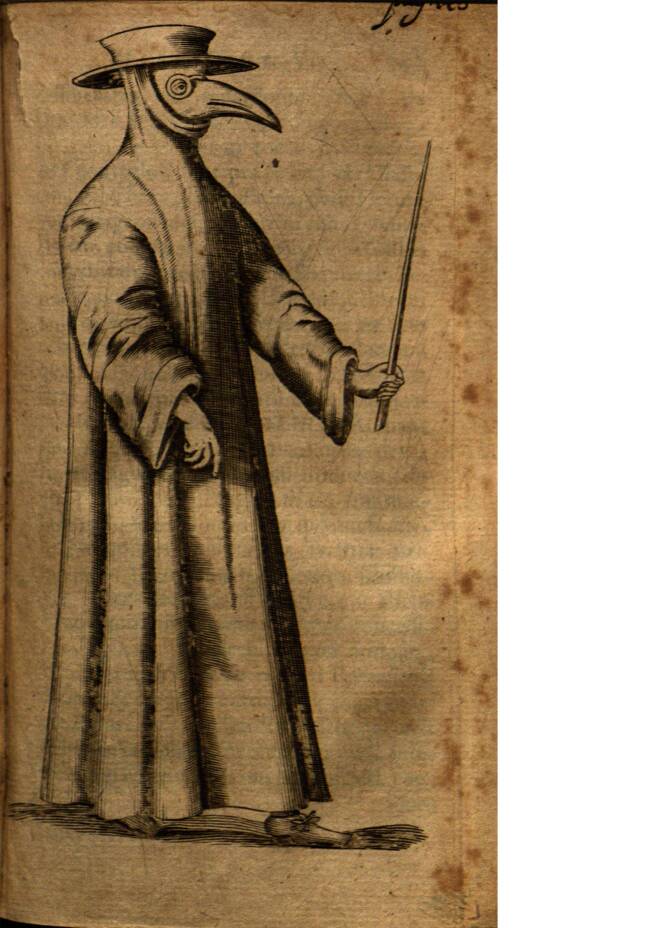

**Figure 3:**
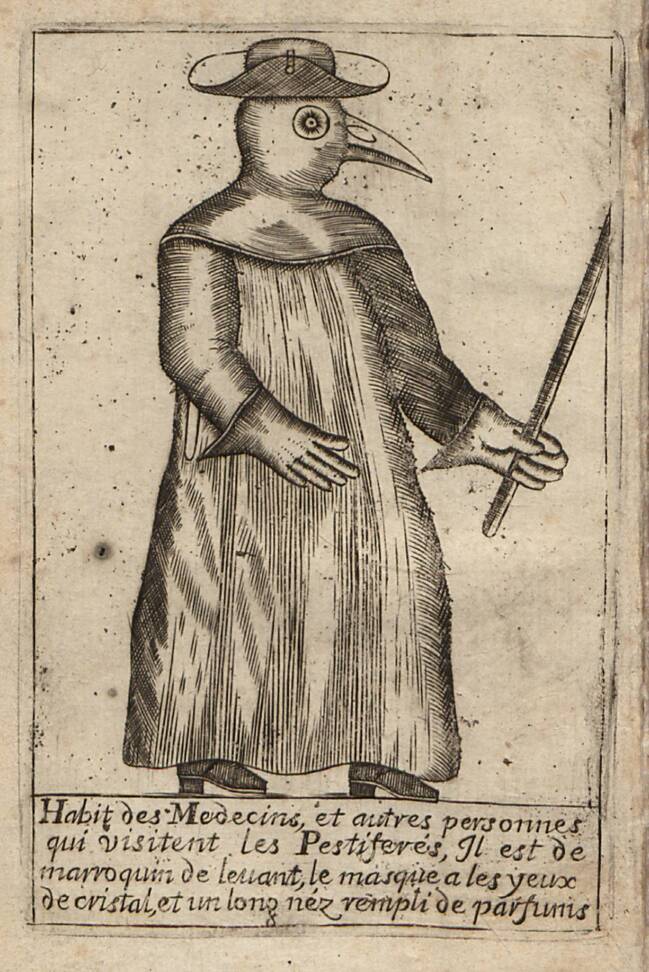

Als Jean-Jacques Manget (1652–1742) rund 60 Jahre später in Genf anlässlich der Pestepidemie von Marseille sein auf Literaturstudien und Briefen basierendes *Traité de la Peste* veröffentlichte, stellte er ihm als Titelkupfer eine weitere Darstellung des Schnabeldoktors voran (Abb. 3). Die Bildlegende lautete: „Gewand der Ärzte, und anderer Personen, welche die Pestkranken besuchen, Es ist aus levantinischem Maroquinleder, die Maske hat Augen aus Kristall, und eine lange Nase voller Duftstoffe (*parfums*).“ (Manget 1721). Diese knappe Erläuterung wird im zweiten Teil des Werkes durch einen ausführlicheren Kommentar ergänzt. Demzufolge sei diese Schutzkleidung keine neue Erfindung, sondern in Italien schon vor langer Zeit bekannt gewesen. Wichtig war Manget der Hinweis, dass der lederne „Schnabel“ zwar nur zwei Nasenlöcher habe, diese aber zum Atmen ausreichten. Die Duftstoffe in seinem Inneren aromatisierten die einströmende Luft und schützten den Arzt so vor dem gefürchteten Pesthauch.

Für das Gewand des Pestarztes wurde weich gegerbtes Ziegenleder verwendet, das durch seine geschlossene, glatte Oberfläche das Anhaften von Contagien noch besser verhindern sollte als Leinen. Dieses Leder war in verschiedenen Varianten im Handel: Das im Osmanischen Reich nach einem geheimen Verfahren hergestellte, besonders hochwertige „Maroquin“ war auch in Rot und anderen Farben erhältlich (Krünitz 1802), das vor allem in Hamburg und Lübeck produzierte „Corduan“ dagegen hatte stets eine schwarze Farbe, was der daraus gefertigten Arztkleidung eine düstere, an Tod und Trauer gemahnende Anmutung verlieh (vgl. Abb. 5; Zedler 1733).

Die beiden Darstellungen weisen, bei aller Ähnlichkeit, doch einige Unterschiede auf: Bei Bartholin (Abb. 2) hat der Pestarzt bloße Hände, während er bei Manget (Abb. 3) Lederhandschuhe trägt, deren Stulpen über die schmal geschnittenen, langen Ärmel gezogen sind. Noch auffallender ist die Ausgestaltung des Kopfschutzes: Bei Bartholin trägt der Pestarzt eine das Gesicht bedeckende Schnabelmaske mit Brille, die an die Figur des Dottore in der Commedia dell’Arte erinnert. Erst durch die Kombination dieser Maske mit dem hochgezogenen Mantelkragen und dem Doktorhut wird die gewünschte Verhüllung des Hauptes erreicht; bei Manget hingegen sind der Schnabel und die Augengläser in eine Haube eingearbeitet, die den ganzen Kopf und die Schultern bedeckt. Alle späteren Abbildungen von Pestärzten basieren auf diesen beiden Varianten. Für die weitere Beschäftigung mit dem Phänomen des Schnabeldoktors bietet sich daher eine typologische Unterscheidung in einen „Maskentyp“ (nach Bartholin 1661) und einen „Haubentyp“ (nach Manget 1721) an.

Die frühesten bekannten Belege für die Anwendung des schnabelartigen Nasenfutterals stammen, wie oben ausgeführt, aus dem 17. Jahrhundert. Sie beziehen sich allerdings nur auf Frankreich und Italien. Für den deutschsprachigen Raum sind keine entsprechenden Quellen nachweisbar.^4^ Für die Zeit davor, also vom „Schwarzen Tod“ des Spätmittelalters bis zum ausgehenden 16. Jahrhundert, lässt sich nur *ex negativo* argumentieren: In den zeitgenössischen Dokumenten und Druckschriften haben sich bislang keinerlei Hinweise auf die Diskussion oder Verwendung dieser auffallenden Schutzkleidung finden können.^5^

## Die „Pestmaske“ des DMMI

Treten wir mit dem neu gewonnenen Wissen um die zeitlich und räumlich begrenzte Präsenz von „Schnabelärzten“ nun mit kritischem Blick vor die Pestarzthaube des DMMI (Abb. 1). Leider lässt sich die Provenienz der Haube nur bis zu dem Kunsthändler in Stuttgart zurückverfolgen, bei dem sie 2002 ersteigert wurde. Hier endet die Spur; wir wissen nicht, ob diese nach Italien oder Frankreich zurückgeführt hätte.

Das Material – leinwandbindiges, ursprünglich wohl imprägniertes Leinen für die Haube und Leder für den Schnabel – entspricht den zeitgenössischen Empfehlungen. Weitere Aufschlüsse ergab das 2013/14 durchgeführte Restaurierungsprojekt, bei dem die Maske von einer auf Textil und Leder spezialisierten Diplom-Restauratorin konservatorisch überarbeitet wurde. Sie unterfütterte und stabilisierte die fadenscheinigen und ausgebrochenen Bereiche, reinigte die Oberfläche und polsterte die Halterung so aus, dass Schnabel, Stoff und Nähte möglichst vom Eigengewicht entlastet wurden. Dabei zeigte sich, dass auch die Machart der Maske eine Datierung auf das 17. oder 18. Jahrhundert zulassen würde. Die Nähte sind im Vor- und Festonstich von Hand gearbeitet; die innere Konstruktion ist relativ aufwendig ausgeführt, mit einem gefütterten Gesichtsschutz aus Leinen, der die Ohren- und Mundpartie bedeckt und möglicherweise über genähte Ösen am Hinterkopf festgezogen werden kann. Eine gesteppte Partie rund um den Kopf dient als Stirnband und stabilisiert den Sitz der Haube auf dem Kopf (Abb. ESM 4).^6^ Die etwa 20 cm lange Nase bietet reichlich Platz für die Aufnahme von Duftstoffen, die über den Kopfraum eingebracht werden können.

Wenn man sich in die Situation des Pestarztes versetzt, der diese Haube beim Krankenbesuch tragen soll, drängen sich zwei Aspekte auf, die an der Alltagstauglichkeit der Konstruktion zweifeln lassen: Zum einen ist der Abstand der Augengläser weiter als normal, so dass man nur eingeschränkt sehen kann; zum anderen hat der Schnabel keine Nasenlöcher, so dass er seinen eigentlichen Zweck, die Parfümierung der Atemluft, verfehlt. Zudem würde man rasch unter Luftnot leiden: Die Poren der Leinwand sind durch die Beschichtung versiegelt, der Tunnelzug in der Halspartie schließt sie nach unten ab, es gibt kaum Frischluftzufuhr. Auch weist die Haube innen keine Gebrauchsspuren auf; es darf also mit gutem Grund an ihrer Authentizität gezweifelt werden. Vielleicht wird die noch ausstehende naturwissenschaftliche Analyse der verwendeten Materialien die endgültige Klärung bringen.

## Im Vergleich: Die „Pestmaske“ des DHM

**Figure 4:**
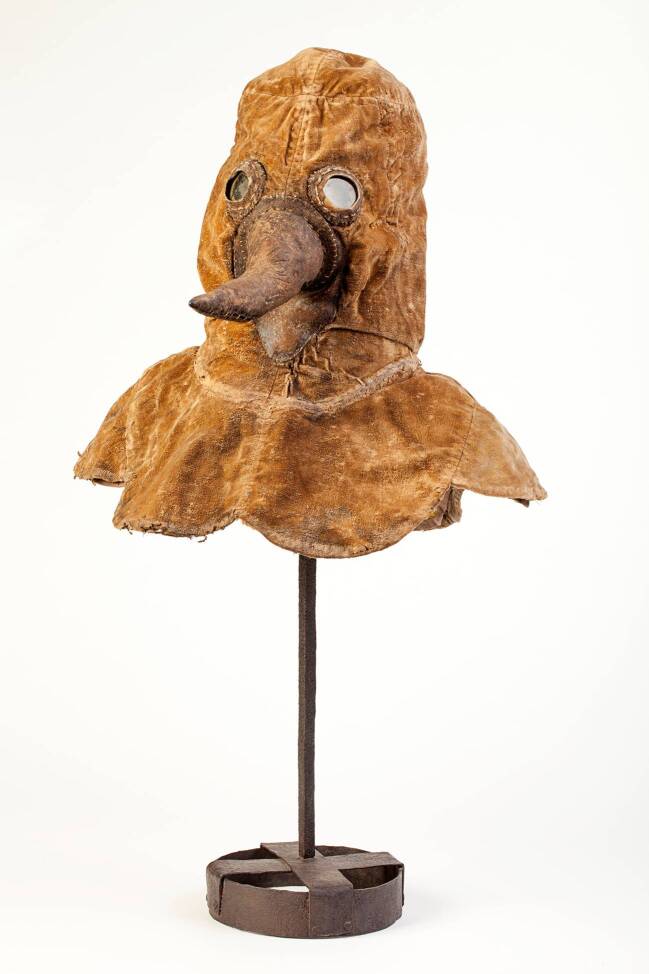

Durch das Entgegenkommen der Kolleginnen vom Deutschen Historischen Museum (DHM) in Berlin wurde es möglich, die zweite uns bekannte Schnabelhaube Deutschlands als Vergleichsobjekt hinzuzuziehen (Abb. 4).^7^

Auch hier endet die Provenienz beim Verkäufer: Die Haube wurde 2006 in einem österreichischen Auktionshaus für die Neugestaltung der Dauerausstellung des DHM erworben. Die mitgelieferte, aber nicht weiter belegte Angabe, dass sie aus Deutschland oder Österreich stamme, scheint angesichts des neuen Forschungsstands wenig glaubwürdig. Die Datierung der Haube auf „1650 bis 1750“ passt durchaus zu Material und Verarbeitung. Auch hier sind die (aus Selenit gefertigten) Augengläser unnatürlich weit gesetzt. Im Gegensatz zu ihrem Ingolstädter Pendant weist sie an der Nasenwurzel beidseits kleeblattförmig eingestanzte „Nasenlöcher“ für die Frischluftzufuhr auf (Abb. ESM 5).

Ein weiterer Unterschied zum Ingolstädter Modell besteht in einem Gitter aus geflochtenen Lederstreifen an der Basis der Nase (Abb. ESM 6). Dieses Gitter erschwerte zwar das Einbringen von Duftschwämmen, Kräutersäckchen oder dergleichen, verhinderte aber zugleich deren Herausrutschen, das für den Träger der Haube sehr unangenehm gewesen wäre; zudem standen geeignete Duftstoffe auch als Balsam oder Öl zur Verfügung.

Was bei dieser Haube jedoch irritiert, ist die Wahl des Stoffes: Sie ist aus Baumwollsamt gearbeitet. Samt aber war ein denkbar ungeeignetes Material für eine Seuchenschutzkleidung, weil das Krankheitsgift gemäß der zeitgenössischen Theorie an seiner Oberfläche besonders leicht haften bleiben konnte. Daran änderte auch das gewachste, ungebleichte Leinengewebe nichts, das als Futterstoff verwendet wurde. Auch wenn die Haube deutliche Gebrauchsspuren aufweist, ist es daher mehr als unwahrscheinlich, dass sie wirklich zu Pestzeiten getragen wurde.

## Fazit: Die Karriere einer Randerscheinung

Der „Schnabeldoktor“ war demnach bestenfalls eine Randerscheinung der Pest, er begleitete erst ihren Abgesang. Seine Präsenz genügte bei weitem nicht, um sich in das kollektive Gedächtnis des europäischen Pesterlebens einzuschreiben. Auch in zeitgenössischen Pestbildern kommt er nicht vor; die Künstler zeigten stattdessen Kranke, Sterbende und Tote (Mollaret & Brossollet 1965). Wie kam es dazu, dass er dennoch zu dem Symbol der Pest schlechthin avancierte?

**Figure 5:**
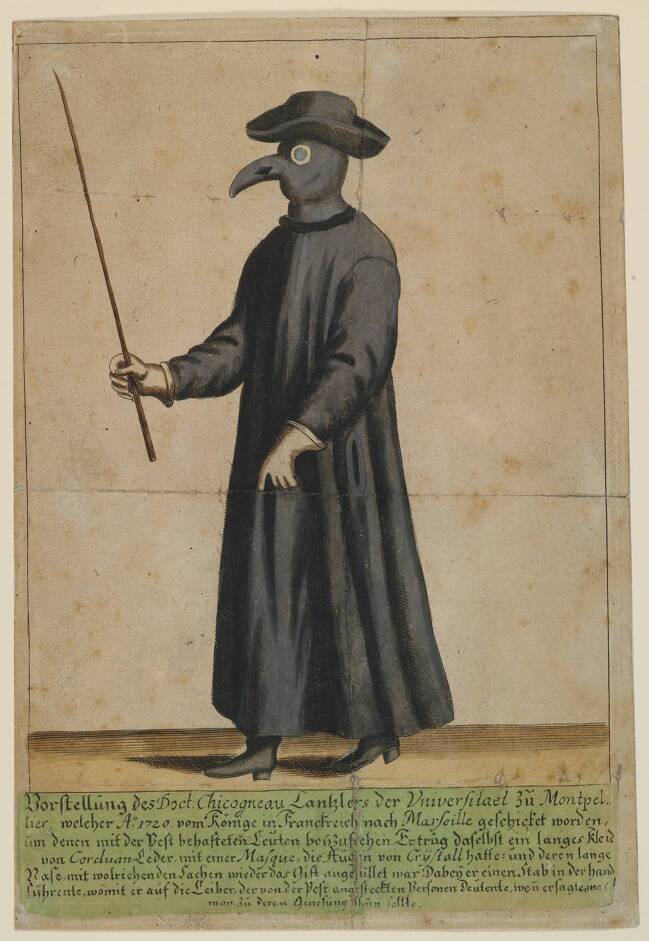

Der Pestarzt mit der Schnabelmaske machte, wenn man so will, eine virtuelle Karriere. Er prägte die Ikonographie der Pest nicht durch seine reale Existenz, sondern durch seine Abbildung in einer Serie von Einblattdrucken, die in den Jahrzehnten um 1700 verbreitet wurden. Als Beispiel sei das im Germanischen Nationalmuseum Nürnberg aufbewahrte, originalkolorierte Blatt angeführt, das den Kanzler der Universität von Montpellier in einem ledernen Schutzgewand bei seinem Einsatz als Pestarzt in Marseille 1720 zeigt (Abb. 5).

**Figure 6:**
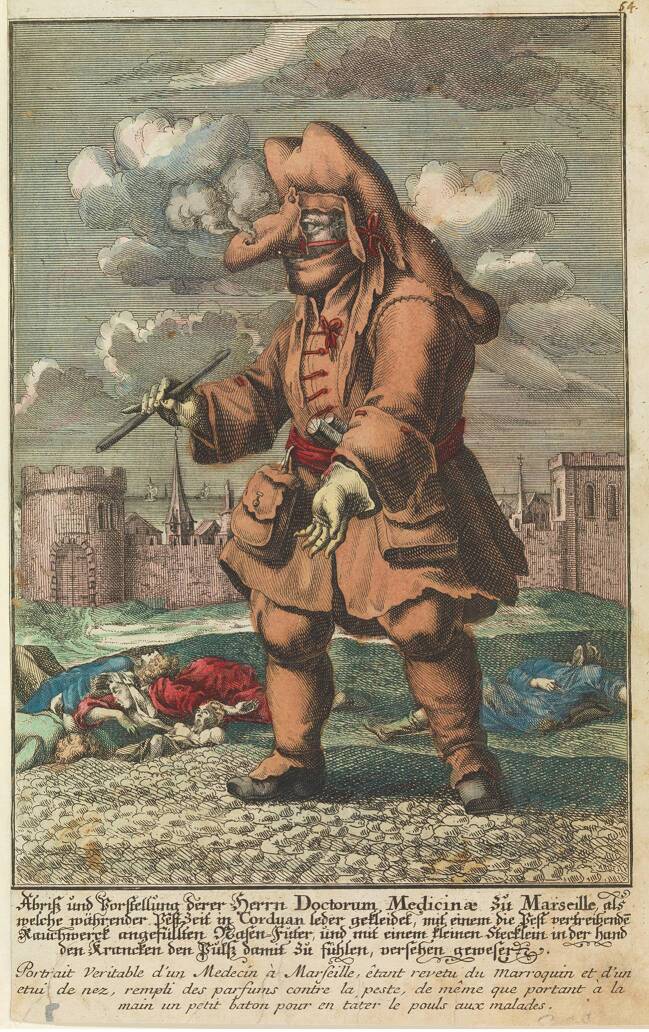

Die Bildunterschriften sind bei diesen Einblattdrucken in deutscher Sprache verfasst, das jeweilige Pestgeschehen spielt sich aber immer außerhalb des deutschen Sprachraums ab, etwa in Südfrankreich (Marseille) oder Italien (Neapel, Rom). Dieser Blick auf die „Pest in der Fremde“ nimmt bisweilen geradezu groteske Züge an (Abb. 6).

Die Blätter stammen wohl überwiegend aus Druckereien in Nürnberg und Augsburg, mithin aus Städten, in denen, wie Annemarie Kinzelbach unlängst zeigen konnte, die Pestfreiheit im 18. Jahrhundert zum Zeichen eines funktionierenden Staats- und Gesundheitswesens wurde (Kinzelbach 2019). Sie können somit auch als ein Instrument der politischen Propaganda der oberdeutschen Reichsstädte verstanden werden.

Die Abgrenzungs- und Überlegenheitsrhetorik dieser Propaganda funktioniert bis heute, wenn auch mit geänderter Zielrichtung. Ursprünglich wurden die Einblattdrucke mit dem Motiv des Schnabeldoktors zur Abgrenzung gegenüber der (vermeintlich schlechter funktionierenden) Gesundheitsverwaltung südeuropäischer Städte in Umlauf gebracht. Heute bedienen sie dagegen das offenbar weit verbreitete Bedürfnis, unser „Heute“ in seiner (vermeintlichen) Überlegenheit von einem nicht näher definierten, dunklen „Früher“ abzusetzen, dessen Medizin von Aberglauben, Magie und ärztlicher Hilflosigkeit bestimmt gewesen sein soll.

Der „Schnabeldoktor“ mit seiner Kombination aus schwarzem Leder, Todesnähe und unscharfem Geschichtsverständnis trifft den Nerv der Zeit; er erlebt gerade seinen zweiten (diesmal digital vermittelten) Medienhype. Die *plague mask* ist in zahllosen Online-Shops als Attribut für Gothic Outfits und Halloween-Verkleidungen erhältlich,^8^ der *plague doctor* begegnet uns in virtueller Form als Bildschirmschoner^9^ und in Computerspielen,^10^er taucht in der „Pest-Strasse“ im Berliner „Dungeon“ auf,^11^ bevölkert Mittelaltermärkte und Kunstgalerien.^12^ Die Anschlussfähigkeit der Pestarzt-Figur ist nicht zuletzt darin begründet, dass sie, bei aller Fremdartigkeit, auf einem uns bekannten Prinzip basiert: der Verhüllung des Körpers zum Schutz vor Ansteckung. Die aktuelle Covid-19-Pandemie hat diesem Prinzip zu einer für uns bislang unbekannten Präsenz verholfen, bis hin zur Diskussion um die Sinnhaftigkeit des Tragens selbstgenähter Mund-Nasen-Masken, die derzeit die Tagesberichterstattung füllt.

## Konsequenzen für die Präsentation im Museum

Die Figur des Pestarztes mit dem schnabelartigen Nasenfutteral ist bislang nur für die Zeit nach 1600 und auch dann nur für den französischen und italienischen Raum belegt. Seine Verwendung für Publikationen oder Ausstellungen mit einem anderen zeitlichen oder regionalen Bezug ist als historisch nicht belegbar abzulehnen. Dies ist auch bei zukünftigen Präsentationen der beiden in Berlin und Ingolstadt aufbewahrten Schnabelhauben zu bedenken; hier bestehen zudem Zweifel, ob die beiden Hauben tatsächlich für die Verwendung in Seuchenzeiten hergestellt wurden.

Für die Neugestaltung der Dauerausstellung in Ingolstadt, deren Eröffnung für September 2020 geplant ist, haben wir uns dafür entschieden, eben diesen Aspekt zum Thema zu machen. Als letztes Objekt des Ausstellungsrundganges stellt es sich den Museumsbesucher*innen unter der Überschrift „Zweifel“ entgegen und präsentiert drei unterschiedliche, sich widersprechende, aber jeweils für sich plausibel argumentierende Objektlegenden. Wir hoffen, unseren Gästen im Museum ebenso wie den Leser*innen dieses Beitrags am Beispiel der Pestarztmaske vor Augen führen zu können, wie wichtig es ist, auch Bildern und Objekten kritisch gegenüber zu treten und bei ihrer Interpretation dieselbe wissenschaftliche Sorgfalt anzuwenden wie bei der Arbeit mit Texten. Im Deutschen Historischen Museum wird man im Rahmen der Neukonzeption der Dauerausstellung ebenfalls diskutieren, in welchem Kontext die dortige Pestarztmaske zukünftig gezeigt werden kann und soll (Bresky & Witt 2020). Denn gerade vertraute Narrative und scheinbar gesetztes Wissen müssen immer wieder aufs Neue hinterfragt, an den Quellen überprüft und gegebenenfalls korrigiert werden, wenn man ein wissenschaftlich fundiertes Geschichtsbild vermitteln will.

## Caption Electronic Supplementary Material





## Footnotes

1 Die hier vorgestellten Forschungsergebnisse zur Authentizität der Pestarztmaske wurden erstmals im Mai 2018 auf dem interdisziplinären Kolloquium „Die Pest“ des LWL-Museums für Archäologie in Herne präsentiert und im Katalog zur dortigen Sonderausstellung „Pest!“ (20.09.2019–10.05.2020) publiziert (Ruisinger 2019).

2 Als Beispiel aus jüngster Zeit sei auf die – handwerklich hervorragend ausgeführte – lebensgroße Figurine eines Pestarztes verwiesen, die in der neuen Dauerausstellung des Stadtmuseums Berlin (Märkisches Museum) im Raum zur Pest des Dreißigjährigen Krieges zu sehen ist.

3 Aus Platzgründen konnten nicht alle Abbildungen in die Printversion aufgenommen werden. Die mit „ESM“ (Electronic Supplementary Material) gekennzeichneten Abbildungen finden Sie unter 10.1007/s00048-020-00255-7.

4 Auch in Zedlers Universallexikon finden sich unter den Lemmata „Pestarzt“ und „Pestbediente“ keinerlei Hinweise auf eine berufsspezifische Schutzkleidung, Zedler (1741a), Zedler (1741b).

5 Dieser Befund wurde bestätigt von Prof. Dr. Dr. Klaus Bergdolt (Köln), Prof. Dr. Fritz Dross (Erlangen), Dr. Annemarie Kinzelbach (München) sowie Prof. Dr. Michael Stolberg (Würzburg), denen ich für die anregenden Gespräche danke.

6 Vgl. Restaurierungsprotokoll der Textilrestauratorin Sonja Müller, Waiblingen.

7 Mein Dank gilt der Kuratorin Dr. Sabine Witt, der Textilrestauratorin Jutta Peschke und Anne-Dorte Krause vom Bildarchiv des DHM für die kollegiale Unterstützung.

8 https://plaguedoctormasks.com [Zugriff 21.5.2020].

9 http://getwallpapers.com/collection/plague-doctor-wallpaper [Zugriff 21.5.2020].

10 https://darkestdungeon.gamepedia.com/Plague_Doctor [Zugriff 21.5.2020].

11 https://www.thedungeons.com/berlin/de/entdecke-das-dungeon/pest-strasse/ [Zugriff 21.5.2020].

12 https://www.bbc.com/news/uk-scotland-edinburgh-east-fife-12934125 [Zugriff 21.5.2020].
