# Supplementary material for: The “Plague Doctor’s Mask” in the German Museum for the History of Medicine, Ingolstadt
Source: NTM. 2020 May 25;28(2):235–52. [Article in German] doi: 10.1007/s00048-020-00255-7 (PMC8156585; doi:10.1007/s00048-020-00255-7)
Supplement: Supplementary file 1 [file 48_2020_255_MOESM1_ESM.pdf]

Die Pestarztmaske im Deutschen Medizinhistorischen Museum Ingolstadt
– ESM (Electronic Supplementary Material) –

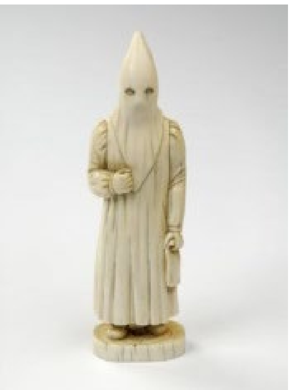

Abb. 2 ESM
Elfenbeinstatuette eines „Pestarztes“,
um 1700 (?) (DMMI, Inv,-Nr. AB/0315)

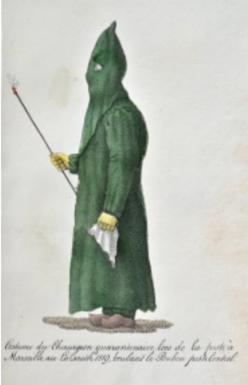

Abb. 3 ESM
Quarantänechirurg am Lazarett von Marseille
(DMMI, Robert 1826, Bd. 2, Anhang)

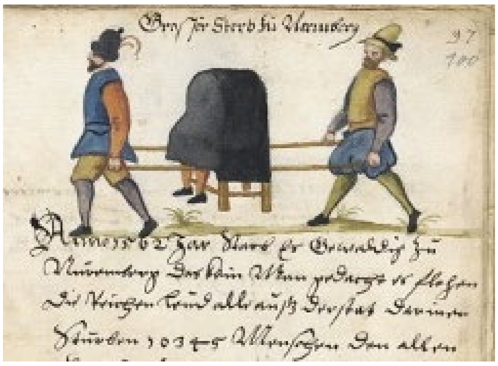

Abb. 4 ESM
Von der Stadt Nürnberg angestellte Träger bringen
1562 einen Pestkranken in das Pesthaus St. Sebastian
vor den Toren der Stadt. (Neubauersche Chronik,
Stadtarchiv Nürnberg, Sign. F1-42-100)

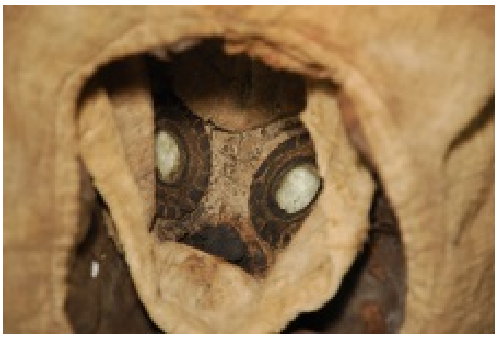

Abb. 7 ESM
Blick von Innen auf den Gesichtsteil der Ingolstädter
Pestarztmaske (Foto: Textil-Restauratorin Sonja Müller,
Waiblingen)

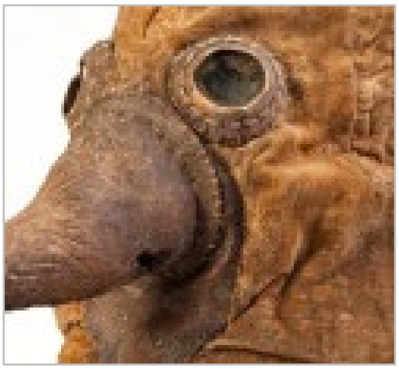

Abb. 9 ESM
Detailansicht der Berliner Pestarztmaske
mit Darstellung der Nasenlöcher
(DHM, Foto: C. Schlegelmilch)

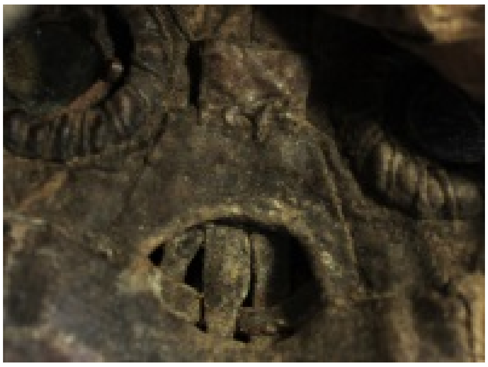

Abb. 10 ESM
Blick von innen auf den Gesichtsteil
der Berliner Pestarztmaske
(DHM, Foto: Jutta Peschke)

Prof. Dr. Marion Maria Ruisinger | Deutsches Medizinhistorisches Museum Ingolstadt
Anatomiestr. 18-20 | 85049 Ingolstadt | marion.ruisinger@ingolstadt.de | www.dmm-ingolstadt.de
